# Why People with More Emotion Regulation Difficulties Made a More Deontological Judgment: The Role of Deontological Inclinations

**DOI:** 10.3389/fpsyg.2017.02095

**Published:** 2017-11-28

**Authors:** Lisong Zhang, Zhongquan Li, Xiaoyuan Wu, Ziyuan Zhang

**Affiliations:** ^1^School of Social and Behavior Sciences, Nanjing University, Nanjing, China; ^2^Institute of Disability Research, Nanjing Normal University of Special Education, Nanjing, China; ^3^Department of Applied Foreign Language Studies, Nanjing University, Nanjing, China

**Keywords:** emotion regulation difficulties, moral judgment, deontological inclinations, utilitarian inclinations, process-dissociation approach

## Abstract

Previous studies have demonstrated the key role of emotion in moral judgment, and explored the relationship between emotion regulation and moral judgment. The present study investigated the influence of individual differences in emotion regulation difficulties on moral judgment. Study 1 examined whether individuals with high emotion regulation difficulties made a more deontological judgment. Study 2 explored the underlying mechanism using a process-dissociation approach, examining whether deontological inclinations and utilitarian inclinations separately or jointly accounted for the association. The results indicated that individuals with high emotion regulation difficulties rated the utilitarian actions less morally appropriate, and one’s deontological inclinations mediated the association between emotion regulation difficulties and moral judgment.

## Introduction

Moral judgment involves an evaluation of one’s or others’ actions on moral acceptability (Avramova and Inbar, 2013; Szekely and Miu, 2015a). Among moral dilemmas in the harm domain, people have to make judgments about which option is more morally acceptable, hurting one person to save a number of others, or not hurting the person regardless of its consequences. For example, in one version of the very famous trolley dilemmas, a runway trolley is out of control and endangers the lives of five workers working on its current track. Participants face two options: to hit a switch to divert the trolley to another track, and to save the five workmen at the cost of the death of one workman on that track, or to do nothing, and to let the trolley cause the deaths of the five workmen (Greene et al., 2004). The preference for the former is regarded as a utilitarian judgment (Mill, 1861/1998), judging mainly on the basis of the consequences of the actions (Elqayam et al., 2017), and the preference for the latter is interpreted as a deontological judgment (Kant, 1785/1959), judging mainly on the basis of pre-existing obligations (Elqayam et al., 2017).

For a long time in the past, some theorists claimed that moral judgment is a pure rational process (e.g., Kohlberg, 1971), whereas others argued the critical role of emotion in moral judgment (Haidt, 2001; Greene and Haidt, 2002). Greene (2007) proposed a dual process model of moral judgment to reconcile these two conflicting views, and argued that both affective and cognitive processes were involved in moral judgments. Empirical evidence suggests that deontological judgment will be made if the negative emotion experienced during moral dilemmas is powerful enough, or the person has limited time or resources to complete deliberative consideration. Otherwise, utilitarian judgment will be made (Greene et al., 2001, 2008, 2009; Cushman et al., 2010).

Moral dilemmas always induce negative emotions such as anger, contempt and disgust in persons who have to make the decisions (Avramova and Inbar, 2013), while people often regulate their emotions in different ways, consciously or unconsciously (Gross, 2013). Increasing evidence indicates the association between emotion regulation and moral judgment (Feinberg et al., 2012; Lee and Gino, 2015; Szekely and Miu, 2015b; Li et al., 2017). Some researchers focused on the strategies used in emotion regulation. For instance, Feinberg et al. (2012) and Szekely and Miu (2015b) both found cognitive reappraisal resulted in more utilitarian judgments, while Lee and Gino (2015) reported expressive suppression led to more utilitarian judgments. However, other scholars started to pay attention to deficits in emotion regulation. Emotion regulation difficulties mean failures in the control and reduction of emotional experience and expression of negative emotions (Cortez and Bugental, 1994). Zhang et al. (2017) pioneered in exploring the impact of individual differences in emotion regulation difficulties on moral judgment in five domains suggested by Moral Foundation Theory (Graham et al., 2011): Harm, Fairness, Authority, Loyalty and Sanctity. Their results indicated that more emotional regulation difficulties were associated with high immorality judgments (or deontological judgments) in all five moral domains, and emotional valence and arousal accounted for the association in the Harm, Fairness, and Sanctity domains.

According to Greene (2007), two kinds of moral inclinations underlying moral judgment, i.e., deontological and utilitarian inclinations, are distinct and independent processes, and are active at the same time. The final moral judgment is determined by the relative strength of deontological and utilitarian inclinations. Stronger utilitarian inclinations lead to utilitarian moral judgments, while stronger deontological inclinations result in deontological judgments. Due to limitation of the traditional data analytic strategy, Zhang et al. (2017) could not tell whether the increased deontological judgment for individuals with high emotion regulation difficulties was caused by an increase in deontological inclinations or a decrease in utilitarian inclinations. Conway and Gawronski (2013) employed a process-dissociation approach proposed by Jacoby (1991) to solve the problem posed by the traditional data analytic strategy, and quantified the strength of deontological and utilitarian inclinations.

We first attempted to replicate the findings in Zhang et al. (2017) on the relationship between individual differences in emotion regulation difficulties and moral judgments. According to Greene’s dual-process model, aversive emotions arise when individuals are faced with moral dilemmas, and then emotion process drives the deontological judgement. In addition, moral dilemmas here focused on the harm theme, and had a higher self-involvement than those in Zhang et al. (2017) selected from a series of moral violating scenarios (Clifford et al., 2015). We could expect that emotion regulation difficulties were positively associated with non-acceptance of utilitarian actions (deontological judgment).

We also investigated the mechanism underlying which increased emotion regulation difficulties may lead to more deontological choices with the process-dissociation approach, so as to figure out whether a decrease in utilitarian judgment was driven by an increased deontological inclination or by a decreased utilitarian inclination. In the dual-process theory of moral judgment, Greene (2007) argued that deontological inclinations are based on emotional reactions to harmful action, while utilitarian inclinations are rooted in a cognitive cost-benefit analysis of the outcome of harmful action. Since deontological inclinations and utilitarian inclinations were independent processes, and the former was closely related to emotional process, while the latter was closely related to cognitive processes (Conway and Gawronski, 2013). Two emotion regulation strategies, cognitive reappraisal and expressive suppression, had no significant effect on utilitarian inclinations, but could significantly reduce deontological inclinations (Lee and Gino, 2015). Similarly, emotion regulation difficulties are more directly related to emotional response. In moral dilemmas, participants experience emotions with more negative valence and higher arousal (Zhang et al., 2017). We hypothesized that deontological inclinations accounted for the relationship between emotion regulation difficulties and moral judgment.

The aim of the present study was to contribute to the understanding of the relationship between emotion regulation difficulties and moral judgment, and to clarify the underlying mechanisms. In Study 1, we examined the relationship between emotion regulation difficulties and moral judgment, and hypothesized that emotion regulation difficulties were positively associated with non-acceptance of utilitarian actions. In Study 2, we further investigated the mechanism by which emotion regulation difficulties resulted in deontological judgments via using a process-dissociation approach, and hypothesized that deontological inclinations uniquely provided an explanation for the association between emotion regulation difficulties and moral judgment.

## Study 1

In Study 1, we examined the relationship between emotion regulation difficulties and moral decision making. Drawing on the dual-process theory, we hypothesized that high emotion regulation difficulties were related to low rating on the appropriateness of the utilitarian action.

### Method

#### Participants

One hundred and sixty-nine undergraduate students who enrolled in a College English course at one university in East China were invited to participate in the study. Due to abnormal responses (e.g., most items were rated with the same numbers), 13 records were removed. Finally, responses from 156 participants were included in later analysis. Most participants were freshmen and sophomores, among which 57% were sophomores. Their age ranged from 18 to 22 years old (*M* = 19.85, *SD* = 0.84), and 51.3% were females. They all received course credit as reimbursement.

#### Measures

##### The difficulties in emotion regulation scale

This measure was developed by Gratz and Roemer (2004) to assess individual differences in emotion regulation difficulties. It consists of 36 items, and each item is rated on a 5-point Likert-type Scale, from 1 (almost never) to 5 (almost always). One sample item is “When I’m upset, I lose control over my behaviors.” A total score is obtained by summing the ratings on all the items. The higher the score, the more difficulties in emotion regulation a person has. The scale demonstrated with good psychometric properties in various studies (e.g., Gratz and Roemer, 2004; Tull et al., 2009; Weinberg and Klonsky, 2009; Berzenski and Yates, 2010). A Chinese version of this scale (Wang et al., 2007) was applied, and the internal consistency computed as *Cronbach alpha* coefficient was 0.91 in the present study.

##### Moral dilemmas

Four moral dilemmas (i.e., Ecologists, Lifeboat, Sophie’s choice, and Submarine) were selected from Greene et al. (2004). Each of the dilemmas described a morally ambiguous situation where the moral agent had to rate how appropriate it is to kill or injure one person in order to save multiple others (utilitarian action). Participants were instructed to read the moral dilemmas, and to assume the role of the moral agent in the scenario. They were also asked to rate the appropriateness of the agent’s utilitarian actions on a 6-point Likert scale (1 = completely inappropriate, 6 = completely appropriate). The internal consistency computed as Cronbach alpha coefficient was 0.70.

#### Procedure

All participants first made their responses to the items assessing emotion dysregulation, and then read and responded to several moral dilemmas, as well as demographic questions, before getting debriefed.

### Results

All data analyses were performed with SPSS 20.0. We first computed descriptive statistics, and then explored the relationship between emotion regulation difficulties and moral judgments with hierarchical multiple regression.

#### Descriptive Statistics

**Table 1** presents the descriptive statistics, including the mean, standard deviation, and Cronbach’s alphas as well as correlations. From the **Table 1**, we can see that emotion regulation difficulties were significantly related to moral judgment. Higher emotion regulation difficulties were related to lower rating on moral appropriateness of the utilitarian action. Besides, there was a significant negative correlation between gender and moral judgment. That is, females rated the utilitarian action as less appropriate than males did.

**Table 1:** Means, standardized deviation, reliabilities, and correlations for major variables.

	*M*	*SD*	1	2	3	4
(1) Age	19.85	0.84				
(2) Gender	0.51	0.50	-0.05			
(3) DERS	89.94	18.07	0.03	-0.01	(0.91)	
(4) Moral judgment	14.37	4.10	-0.05	-0.16^∗^	-0.27^∗∗^	(0.70)


**N* = 156. Gender: 0 = male, 1 = female. DERS = the Difficulties in Emotion Regulation Scale. ^∗^*p* < 0.05, ^∗∗^*p* < 0.01.*

#### Regression Analysis

Using a hierarchical multiple regression analysis, we tested our hypothesis that individuals with more emotion regulation difficulties rated the utilitarian actions less morally appropriate. In the model, moral judgment was treated as the dependent variable, and gender was entered into the first block as a control variable, and the DERS score into the second block. The result (see **Table 2**) indicated that the female rated the utilitarian actions less morally appropriate, β = -0.16, *p* < 0.05. The result also indicated that after controlling the effect of gender, the DERS score could still significantly predicted their moral judgment, β = -0.28, *p* < 0.001. Higher emotion regulation difficulties were associated with the less acceptable judgment of the utilitarian actions.

**Table 2:** Summary of hierarchical multiple regression analyses for predicting moral judgment.

	Model 1		Model 2	
		
Variables	*B* (*SE*)	β	*B* (*SE*)	β
Gender	-1.31 (0.65)	-0.16^∗^	-1.34 (0.63)	-0.16^∗^
DERS			-0.06 (0.02)	-0.28^∗∗∗^
*R*^2^	0.03^∗^		0.10^∗∗∗^	


*^∗^*p* < 0.05, ^∗∗∗^*p* < 0.001.*

### Discussion

In this study, we focused on the relationship between emotion regulation difficulties and moral judgment. The results revealed that individuals with more emotion regulation difficulties rated the utilitarian actions less morally appropriate. The finding supported our hypothesis that higher emotion regulation difficulties was associated with more deontological judgments. The results were also consistent with Zhang et al. (2017) that higher emotion regulation difficulties were related to immortality rating on moral vignettes in harm area. They interpreted the result as the negative emotions induced by moral dilemmas could not be regulated efficiently by individuals with more emotion regulation difficulties (both in valence and arousal), and resulted in deontological judgments. However, it is still unclear whether an increase in deontological inclinations, or a decrease in utilitarian inclinations resulted in less utilitarian judgments of individuals with higher emotion regulation difficulties. We would like to further examine the roles of deontological inclinations and utilitarian inclinations in the association between emotion regulation difficulties and moral judgment using a process dissociation approach.

## Study 2

Study 1 showed that individuals with high emotion regulation difficulties made more deontological judgments. Their preference for deontological choices might be caused by an increase in deontological inclinations, or a decrease in utilitarian inclinations or both. To clarify the effect, in this study, we examined the underlying mechanism using a process-dissociation approach (Conway and Gawronski, 2013).

### Method

#### Participants

Based on the total scores of the DRES in Study1, we selected 30 students at the high end as well as 30 students at the low end, and invited them to attend Study 2. Among the 60 participants, their age ranged from 18 to 22 years old (*M* = 19.93, *SD* = 0.83), and 51.7% were males. They received course credit as reimbursement.

#### Measures

##### Moral dilemmas

Four pairs of moral dilemmas (Crying baby, Abortion, Vaccine Policy, and Relationship) were carefully selected from Conway and Gawronski (2013). In each pair of moral dilemmas, both a congruent version and an incongruent version were included. For the incongruent version, deontological inclinations and utilitarian inclinations compete with each other. That is, the outcome of utilitarian actions outweigh the harm caused by the action, but the action violates deontological moral principles. For example, in the Crying Baby dilemma, it is acceptable to save yourself and the other townspeople from being killed over the baby’ life according to the utilitarian principle, but it is unacceptable to smother the baby according to the deontological principle. For the congruent version, deontological inclinations and utilitarian inclinations are in agreement with each other. That is, the outcome of utilitarian actions doesn’t outweigh the harm caused by the action, and the action would not be acceptable by either deontological or utilitarian principles. For example, in the Crying Baby dilemma, the action of smothering the baby to save yourself and the others from laboring in the mine violates both deontological and utilitarian principles. Two moral dilemmas in each pair were separately presented with other pairs. Participants first read each moral dilemma scenario, then make a judgment on the moral appropriateness of the agent’s utilitarian action, appropriate or inappropriate.

### Results

The choice of “appropriate” was recorded as 1 and the choice of “inappropriate” was recorded as 0. We first calculated the proportion of appropriate response for each participant in both incongruent and congruent dilemmas. Utilitarian action was judged as appropriate 64% of the time (*SD* = 21%) among incongruent dilemmas, while it was judged as acceptable 45% of the time (*SD* = 23%) for the congruent dilemmas. We also summed the choices on both incongruent and congruent moral dilemmas. Utilitarian action was rated as more acceptable in incongruent dilemmas (*M* = 2.56, *SD* = 0.86) than that in congruent dilemmas (*M* = 1.81, *SD* = 0.92), *t*(58) = 7.57, *p* < 0.001.

#### Moral Judgment Analysis

Using a hierarchical multiple regression analysis, we tested our hypothesis that individuals with more emotion regulation difficulties rated the utilitarian actions less morally appropriate in incongruent moral dilemmas. We treated the proportion of appropriate response as the dependent variable, and gender was entered into the first block as a control variable, and the DERS score into the second block. The result (see **Table 3**) suggested that after controlling the effect of gender (β = -0.23, *p* = 0.055), the DERS score could still significantly predict their moral judgment, β = -0.33, *p* < 0.05. Higher emotion regulation difficulties were associated with less appropriate judgments of the utilitarian actions.

**Table 3:** Summary of hierarchical multiple regression analyses for predicting moral judgment.

	Model 1		Model 2	
		
Variables	*B* (*SE*)	β	*B* (*SE*)	β
Gender	-0.10 (0.05)	-0.24	-0.10 (0.05)	-0.23
DERS			-0.003 (0.001)	-0.33^∗∗^
*R*^2^	0.06		0.20^∗∗^	


*^∗∗^*p* < 0.01.*

#### Process-Dissociation Analysis

We adopted the same procedure as Conway and Gawronski (2013) did to compute the process-dissociation scores of utilitarian and deontological inclinations. We first calculated for each participant the probability of rejecting utilitarian actions as acceptable in both congruent and incongruent dilemmas, respectively. And then we computed the utilitarian (U) and deontological (D) parameters. These parameters represented the strength of utilitarian and deontological inclinations, and were standardized before further analysis. The results of independent *t*-tests showed that deontological inclination was significantly higher for the participants with high emotion regulation difficulties (*M* = 0.30, *SD* = 1.23) than those with low emotion regulation difficulties (*M* = -0.30, *SD* = 0.59), *t*(40.01) = 2.39, *p* < 0.05. On the other hand, utilitarian inclinations did not differ significantly between participants with high emotion regulation difficulties (*M* = -0.07, *SD* = 1.07) and those with low emotion regulation difficulties (*M* = 0.07, *SD* = 0.94), *t*(57) = -0.56, *p* = 0.58.

### Discussion

In this study, we further clarified the mechanism underlying the relationship between emotion regulation difficulties and moral judgment. The results indicated that compared to individuals with less emotion regulation difficulties, individuals with more emotion regulation difficulties had more deontological inclinations, and rated the utilitarian actions less morally appropriate. The findings were consistent with our hypothesis that emotion regulation difficulties were selectively related to deontological inclinations while leaving utilitarian inclinations unaffected. The findings were also consistent with previous studies (Conway and Gawronski, 2013; Lee and Gino, 2015). Conway and Gawronski (2013) found that enhanced empathy increased deontological inclinations with utilitarian inclination being unaffected. Similarly, Lee and Gino (2015) results indicated that one’s reduced deontological inclinations mediated the relationship between emotion regulation strategies and moral decision making other than utilitarian inclinations.

## General Discussion

In the two studies, we investigated the association between emotion regulation difficulties and moral judgment, and also the mechanism underlying the association. We found that higher emotion regulation difficulties were associated with less acceptance of utilitarian actions, and increased deontological inclinations accounted for the relationship. High emotion regulation difficulties led to increased deontological inclinations, and resulted in lower preferences for utilitarian actions. These findings supported our hypotheses, and were also consistent with several previous studies (e.g., Conway and Gawronski, 2013; Lee and Gino, 2015; Zhang et al., 2017).

Previous theories and empirical studies have indicated the critical role of emotion in moral judgment (e.g., Valdesolo and DeSteno, 2006; Strohminger et al., 2011; Avramova and Inbar, 2013). Several studies have also investigated the relationship between different emotion regulation strategies and moral judgment (e.g., Szekely and Miu, 2015b). However, the relationship between emotion dysregulation and moral judgment has not been paid enough attention so far (Szekely and Miu, 2015a). Zhang et al. (2017) first examined the impact of individual difference in emotion regulation difficulties with a set of standardized moral scenarios. Their study implied that emotion regulation difficulties could significantly predict immorality judgment in all five moral domains. The present research replicated their findings among high conflicting and high self-involved moral dilemmas in the Harm domain, and found individuals with high emotion regulation difficulties rated the utilitarian actions less appropriate.

The present research also clearly clarified the roles of utilitarian and deontological inclinations in the association between emotion regulation difficulties and moral judgment. It indicated that emotion regulation difficulties selectively increased deontological inclinations, and then led to less utilitarian choices. The finding was consistent with dual process theory (Greene, 2007) and empirical studies (Conway and Gawronski, 2013; Lee and Gino, 2015). Greene (2007) suggested that deontological inclinations were based on emotional response to harmful actions, while utilitarian inclinations were related to cognitive analysis about cost and benefit. Conway and Gawronski (2013) found that enhanced empathy selectively increased deontological inclinations with utilitarian inclinations being unaffected, while cognitive load selectively reduced utilitarian inclination, with deontological inclinations being unaffected. Lee and Gino (2015) found that both cognitive reappraisal and expressive suppression selectively reduced deontological inclinations, with utilitarian inclinations being unaffected. Emotion regulation difficulties have a more direct relationship with emotional reactions, and individuals with high emotion regulation difficulties will experience more negative and higher aroused emotions (Zhang et al., 2017).

There are several promising directions for future research. First, due to limitations of the correlational design, we could not make a clear causal inference about the association between emotion regulation difficulties and moral judgment. Experimental manipulation of emotion regulation difficulties will resolve this problem to some extent (Lavender et al., 2017). Second, we focused on the investigation of moral dilemmas within the Harm domain -only. Extending the exploration to other domains will contribute to the literature of both Moral Foundations Theory and Dual Process Theory. Third, the samples here were college students in China, thus the generalizability of the results to other groups with different ages and cultural backgrounds should be performed with cautions. For example, Pellizzoni et al. (2010) found utilitarian reasoning was also present in children. In addition, Michelin et al. (2010) reported that a pattern of saving more persons emerged earlier among children with Slovenian-Italian linguistic and cultural background than that among children with Italian monolingual children, and was more salient among Slovenian-Italian adults than that among Italian-only speakers. Fourth, the materials we used in present research were hypothetical moral dilemmas. Virtual reality presentation of moral dilemmas will have more ecological validity, and will be better in predicting individuals’ behaviors (Patil et al., 2014). Finally, Elqayam et al. (2015, 2017) proposed a processing model for deontic introduction to explain the process of moral judgment, and found deontic introduction was only related to utilitarian moral judgment. It would be interesting to explore the role of deontic introduction in the association between emotion regulation difficulties and moral judgment.

Notwithstanding these limitations, the current research revealed the relationship between emotion regulation difficulties and moral judgment, as well as the mechanism underlying the association. Emotion regulation difficulties were negatively associated with the endorsement of utilitarian actions. The association could be accounted for by increased deontological inclinations. That is, emotion regulation difficulties affects the deontological inclinations rather than the utilitarian inclinations. It supported the independence of deontological and utilitarian inclinations to moral judgments (Conway and Gawronski, 2013).

## Ethics Statement

This study was carried out in accordance with the recommendations of Ethical Conduct in Human Research by the Institutional Review Board of Department of Psychology, Nanjing University, with written informed consent from all subjects. All subjects gave written informed consent in accordance with the Declaration of Helsinki. The protocol was approved by the Institutional Review Board of Department of Psychology, Nanjing University.

## Author Contributions

All authors conceived, designed, and conducted the studies. ZL conducted the statistical analyses. LZ, ZL, and XW wrote the first draft. All authors revised the final manuscript.

## Conflict of Interest Statement

The authors declare that the research was conducted in the absence of any commercial or financial relationships that could be construed as a potential conflict of interest.
